# Serum metabolomics identifies metabolic changes in obese cats fed enzymatically hydrolyzed poultry byproduct meal

**DOI:** 10.1093/jvimsj/aalaf075

**Published:** 2026-01-21

**Authors:** Leonardo de Andrade Príncipe, Pedro Henrique Marchi, Carolina Dantas Micheletti, João Marcos Bovetto de Campos Valim, Nara Regina Brandão Cônsolo, Raquel Silveira Pedreira, Juliana Toloi Jeremias, Gabriel Henrique Ribeiro, Luiz Alberto Colnago, Julio César de Carvalho Balieiro, Thiago Henrique Annibale Vendramini

**Affiliations:** Pet Nutrology Research Center, School of Veterinary Medicine and Animal Science, University of Sao Paulo, Pirassununga 13635-900, Brazil; Pet Nutrology Research Center, School of Veterinary Medicine and Animal Science, University of Sao Paulo, Pirassununga 13635-900, Brazil; Pet Nutrology Research Center, School of Veterinary Medicine and Animal Science, University of Sao Paulo, Pirassununga 13635-900, Brazil; Department of Nutrition and Animal Production, School of Veterinary Medicine and Animal Science, University of São Paulo, Pirassununga 13635-900, Brazil; Department of Nutrition and Animal Production, School of Veterinary Medicine and Animal Science, University of São Paulo, Pirassununga 13635-900, Brazil; Nutrition Development Center, Grandfood Industria e Comercio LTDA (Premier Pet), Dourado 13590-000, Brazil; Nutrition Development Center, Grandfood Industria e Comercio LTDA (Premier Pet), Dourado 13590-000, Brazil; Brazilian Agricultural Research Corporation (Embrapa – CNPDIA), Sao Carlos 13560-970, Brazil; Brazilian Agricultural Research Corporation (Embrapa – CNPDIA), Sao Carlos 13560-970, Brazil; Pet Nutrology Research Center, School of Veterinary Medicine and Animal Science, University of Sao Paulo, Pirassununga 13635-900, Brazil; Pet Nutrology Research Center, School of Veterinary Medicine and Animal Science, University of Sao Paulo, Pirassununga 13635-900, Brazil

**Keywords:** energy metabolism, lipolysis, metabolites, obesity

## Abstract

**Background:**

Obesity in cats is a complex metabolic condition, and understanding its metabolic processes is essential for gaining new insights into nutrition.

**Hypothesis/Objectives:**

Investigate the effects of enzymatically-hydrolyzed poultry byproduct meal (EHPM-c) on the serum metabolomic profile of obese cats.

**Animals:**

Eighteen adult, neutered, obese domestic cats were enrolled in the study. All subjects underwent comprehensive veterinary evaluations to confirm overall health and rule out concurrent systemic diseases, ensuring a homogenous study population.

**Methods:**

Cats were randomized into 2 groups and fed for 45 days with isonutritive diets containing either 30.8% conventional poultry byproduct meal (CPM-c) with 0.0% EHPM-c, or 17.0% CPM-c with 12.0% EHPM-c. After a 30-day diet standardization period, evaluations were performed at baseline (T0) and 45 days (T45) after consumption of the experimental diets. Metabolic spectra were obtained using nuclear magnetic resonance spectroscopy and analyzed using MetaboAnalyst®.

**Results:**

Principal component analysis did not identify differences in the overall metabolite profiles between the groups, but discriminant analysis identified changes in the intensities of valine, acetate, 1-methylhistidine, and glycerol in the test group after 45 days. Additionally, pathway analysis indicated a significant effect on propanoate metabolism, ethanol degradation, fatty acid biosynthesis, and glycerolipid metabolism in the test group.

**Conclusions and clinical importance:**

These findings suggest enhanced fat mobilization and improved utilization of branched-chain amino acids, potentially benefiting energy metabolism in obese cats.

## Introduction

Obesity in cats is a globally prevalent condition and is one of the most complex metabolic disorders in cats, with its metabolic effects still not fully understood.^1–4^ Although its cause is multifactorial, obesity arises primarily from nutritional imbalance, resulting from increased caloric intake and decreased energy expenditure, which together creates a positive energy balance and leads to excessive fat accumulation.^5^ This condition predisposes cats to comorbidities such as diabetes mellitus,^6–8^ dyslipidemia,^9^ dermatologic conditions,^10^ lower urinary tract disease,^11^^,^^12^ and cardiovascular disorders,^13^ and is associated with important alterations in the serum metabolome. Typical findings include higher triglycerides and ketone concentrations, decreased glycine, serine, and kynurenine concentrations, altered phospholipids, and lower creatinine markers that distinguish obese from lean cats.^14–16^

Energy restriction remains the central axis of obesity treatment through a weight loss program and is essential to reverse excessive fat accumulation, although it presents several challenges. A previous study^17^ suggested an optimal weight loss rate of 0.5%–1% per week, because rapid restriction promotes muscle loss instead of fat.^18^ In this context, weight loss diets with higher inclusions of proteins, essential amino acids, fatty acids, and micronutrients are necessary to balance fat reduction with muscle preservation.^14^^,^^19^^,^^20^

Enzymatically hydrolyzed poultry byproduct meal (EHPM-c) has emerged as an innovative component. Enzymatic hydrolysis breaks proteins into smaller peptides, enhancing digestibility, amino acid absorption, and decreasing allergenic potential.^21^^,^^22^ This ingredient also offers a highly bioavailable amino acid profile, because hydrolyzed proteins are rapidly digested and absorbed, increasing amino acid availability compared with intact proteins.^23–24^ By cleaving peptide bonds, enzymatic hydrolysis minimizes heat-induced damage and enhances gastrointestinal absorption.^20^ Moreover, poultry byproduct hydrolysates release bioactive peptides with antihypertensive, antioxidant,^25^^,^^26^ euglycemic,^27^ hypoallergenic,^22^ and eubiotic properties.^28^

Peptides such as isoleucine-lysine-tryptophan, leucine-lysine-alanine, leucine-lysine-proline, and tyrosine-tyrosine-arginine-alanine from poultry byproducts show biological activity in humans.^29^ Proteolytic enzyme and hydrolysis methods define the peptide profile and its functional properties.^20^ Given the potential systemic effects of these compounds, additional studies are warranted to understand how such ingredients influence metabolic pathways in companion animals. Evidence from other species shows that dietary hydrolyzed proteins and bioactive peptides affect metabolism through enhanced amino acid availability and signaling peptide activity regulating key metabolic enzymes.^25^^,^^30–32^

Metabolomics enables the comprehensive characterization of small molecules in biological samples, providing a systemic view of physiology closely linked to phenotype.^33^ It has been applied to identify disease mechanisms, classify metabolic states, and assess treatment effects. Serum, rich in metabolites reflecting systemic metabolism, is widely validated for such analyses.^34^ In cats, serum metabolomics helps identify metabolic changes from dietary interventions, especially in energy metabolism.^14^^,^^35^^,^^36^ Its use is expanding in both disease and nutritional studies.^37–40^

Alterations in lipid and amino acid metabolism are central to obesity in cats.^14–16^ Obese cats have increased triglycerides, glycerol, and lysophosphatidylcholines (LPC C16:1 and LPC C20:4), indicating dysregulated lipid metabolism and oxidative stress.^14^^,^^15^ Serine concentrations are increased, whereas branched-chain amino acid (BCAA) concentrations remain stable, differing from omnivorous species.^14^^,^^15^

During caloric restriction, long-chain acylcarnitines increase, lipid metabolites decrease, and ketone bodies (3-hydroxybutyrate and acetoacetate) increase, reflecting higher fat utilization.^15^ Meanwhile, glycine, alanine, and glutamine concentrations increase, suggesting muscle protein catabolism for energy.^14^^,^^15^

Given the strict carnivorous nature of cats and high protein needs, understanding these adaptations is key to formulating diets that favor fat loss without compromising lean mass. Although several studies have addressed metabolic alterations in obese cats, further metabolomic research is needed to assess novel dietary ingredients supporting safe, effective weight loss.^15^^,^^41^^,^^42^ Therefore, we investigated the effects of EHPM-c on the serum metabolomic profile of obese cats.

## Materials and methods

Our study was carried out in agreement with the Ethical Principles in Animal Research established by the Ethic Committee on Animal Use of the School of Veterinary Medicine and Animal Science at the University of Sao Paulo (CEUA/FMVZ). The study was approved under protocol number 8609280422. The experiment was conducted at the Pet Nutrology Research Center (CEPEN Pet) of the Animal Nutrition and Production Department of the School of Veterinary Medicine and Animal Sciences of University of Sao Paulo (FMVZ/USP), in the city of Pirassununga, Sao Paulo, Brazil.

### Study design

Eighteen client-owned male (*n* = 12) and female (*n* = 6) mixed-breed, neutered cats, with a mean age of 8.46 ± 0.69 years and a mean body condition score (BCS) of 8 to 9/9 (BCS 8, *n* = 7; BCS 9, *n* = 9), clinically healthy and without associated comorbidities, were included (Table S1).

The BCS of the cats was assessed using a 9-point,^43^ a validated method widely used in clinical practice. Visual inspection and palpation of ribs and lumbar, and abdominal regions was performed, focusing on subcutaneous SC fat, waist definition, and abdominal tuck.^43^ Three veterinary nutritionists independently assessed each cat, and final scores were assigned by consensus. Body weight and BCS were measured every 15 days to maintain BCS between 8 and 9/9 and adjust food quantity when necessary.

The animals’ health status was assessed before enrollment using physical examination and clinical nutritional history taken by a veterinarian. Guardians provided medical and dietary history, vaccination and deworming records, and negative feline immunodeficiency virus and feline leukemia virus test results. Fecal examinations confirmed parasite absence. The physical examination included cardiac and pulmonary auscultation, blood pressure, temperature, capillary refill time, mucous membrane color, and abdominal and lymph node palpation. Cats with abnormalities were excluded and referred for care.

Each selected cat had a CBC and serum biochemical profile performed (albumin, glucose, total protein, urea, creatinine, alkaline phosphatase, cholesterol, triglycerides, aspartate aminotransferase [AST], γ-glutamyl transferase [GGT], and alanine aminotransferase [ALT]; Table S2). The history and examination excluded comorbidities such as urinary, respiratory and dermatologic disorders, or recent drug or supplement use. None of the cats received probiotics, prebiotics, or symbiotics that could affect metabolism. Owners were instructed to feed only the study diet and no treats or other foods; compliance was monitored. These steps ensured obesity resulted solely from a positive energy balance.

The experimental design used was randomized, with the animals randomly distributed into two experimental groups: control group (CG; 6 males and 3 females) and test group (TG; 6 males and 3 females), which underwent different treatments that occurred simultaneously.

The study lasted 77 days and was divided into two periods. The first 30 days corresponded to the diet stabilization period, during which all cats were fed the same commercial diet (PremieR® Sterilized Cats 7 to 11 Years Chicken Flavor) without the inclusion of hydrolyzed meal. This period aimed to standardize the animals’ metabolism before initial blood collection and randomization into experimental groups.

On day 31, a 5 mL blood sample was collected by jugular venipuncture for metabolomic analyses. From days 32 to 76, the second period of 45 days began, during which the cats were fed either the conventional poultry byproduct meal (CPM-c) or EHPM-c diet. Finally, on day 77, additional blood samples were collected. Sixteen cats completed the clinical trial (GC, 7 cats; TG, 9 cats), whereas two control group cats were excluded because owner non-compliance with study protocols.

### Diets

Two experimental dry diets were extruded: a control diet containing 30.80% CPM-c and a test diet containing 17.07% CPM-c and 12.00% EHPM-c (Table 1). The estimated daily energy intake for each cat was 130 kcal × (body weight)^0.4^ per day for maintenance.^44^

**Table 1:** Ingredient composition (%) and chemical composition in dry matter (% DM) and g/1000 kcal of metabolizable energy of experimental foods.

	**Diets**			
Nutrients	Chemical composition (% DM)		g/1000 kcal ME	
	Control (CPM-c)	Test (EHPM-c)	Control (CPM-c)	Test (EHPM-c)
**Crude protein**	33.00	33.00	78.5	78.5
**Ethereal extract**	15.00	15.00	35.7	35.7
**Non-nitrogenous extractives**	34.03	34.31	81.0	81.6
**Mineral matter**	5.83	4.79	13.9	11.4
**Crude fiber**	1.40	1.40	3.3	3.3
**Calcium**	1.09	0.75	2.6	1.8
**Phosphorus**	0.91	0.67	2.2	1.6
**Metabolizable energy (kcal/kg)**	4.203	4.203	4.203	4.203

Ingredients: Conventional poultry byproduct meal (CPM-c); Hydrolyzed poultry byproduct meal (EHPM-c); Corn gluten (60% protein); Rice crumb; Beet pulp; Palatalize powder; Poultry offal oil; Fish oil; Potassium chloride; Sodium chloride (common salt); Acidifying additive; Taurine; Mineral and vitamin premix; DL methionine 99%; Synthetic antioxidant.

Composition of mineral and vitamin premix: vitamin A: 38000 IU/kg, Vitamin B1: 38.27 mg/kg, Vitamin B12: 4800mcg/kg, Vitamin B2: 19.63 mg/kg, Vitamin B6: 27.87 mg/kg, Vitamin D3: 171000 IU/kg, Vitamin E: 535,250 mg/kg, Vitamin K: 2.38 mg/kg, Pantothenic Acid: 49,290 mg/kg, Folic Acid: 2,480 mg/kg, B.H.T.: 5000 mg/kg, Biotin: 1.52 mg/kg, Copper: 14.84 mg/kg, Choline: 5.157 g/kg, Iron: 168.87 mg/kg, Iodine: 1.87 mg/kg, Manganese: 17.41 mg/kg, Niacin: 109.50 mg/kg, Selenium: 0.5 mg/kg, Zinc: 167.45 mg/kg.

Antioxidant composition: Butylated hydroxytoluene (BHT): 100 g/kg, Citric acid: 35 g/kg, Butylated hydroxyanisole (BHA): 10 g/kg.

### Metabolomic analysis

Blood samples from all cats were collected after a fasting period of 12 hours in 10 mL tubes without anticoagulant (BD Vacutainer®, São Paulo, SP, Brazil), following the recommendations described previously.^34^ Immediately after collection, samples were centrifuged at 2000 × *g* for 15 min at 4°C. The serum (supernatant) then was aliquoted into 2 mL Eppendorf tubes, properly labeled, and stored at −80°C until macromolecule extraction. For macromolecule removal, 3 kDa centrifugal filters (Amicon® Ultra-0.5, Merck Millipore Ltd., Ireland) were used. Before use, filters were washed according to the manufacturer’s instructions with 500 μL of Milli-Q water, centrifuged at 14 000 × *g* for 5 min at 4°C. This process was repeated 5 times. Residual water then was removed by reverse centrifugation at 7500 × *g* for 60 s at 4°C. Subsequently, 500 μL of serum were loaded into the filters and centrifuged at 14 000 × *g* for 30 min at 4°C. The filtrates were collected and stored at −80°C until nuclear magnetic resonance (NMR) analysis, following the same protocol described previously.^45^

For all samples, an aliquot of 200 μL was vacuum centrifuged overnight to evaporate the aqueous content of the sample, because the extracts were prepared in an aqueous medium (SpeedDry RCV 2-18 CD plus, Christ; Osterode am Harz, GER). For each sample, the remaining pellet was solubilized in 550 μL of phosphate buffered saline (PBS; 0.1 mM; pD = 7.4) containing 0.5 mM of d3-(trimethylsilyl) − 2,20,3,30-tetradeuteropropionic acid (TMSP-d4; Cambridge Isotopes, Leicestershire, UK) used as NMR internal standard. Subsequently, a volume of 550 μL of each sample was transferred to standard 5 mm NMR tube for NMR measurements.

^1^H NMR spectra were acquired from a 14.1 T (600 MHz for hydrogen frequency) Bruker spectrometer (Bruker Corporation, Karlsruhe, Baden-Wurttemberg, Germany), model Avance III HD, equipped with a BBO 5-mm probe., using temperature 298.15 K. All ^1^H NMR spectra were acquired by means pulse sequence with H_2_O pre-saturation signal, named by Bruker as noesypr1d pulse sequence, assuming the following acquisition parameters: a 90° pulse duration of 15.108 μs, a recycle delay (d1) of 4 s, 106 k data points, 256 scans, an acquisition time of 4.51 s, and a spectral width of 20.02 ppm, 5 ms mixing time (d8) and 4 dummy scans.

Spectra were processed using Chenomx NMR Suite Professional version number 10 software (Chenomx Inc., Edmonton, AB, Canada): phasing and baseline correction were performed, and an exponential line broadening of 0.3 Hz was applied. Spectra were referenced to 2,2-dimethyl-2-silapentane-5-sulfonate (DSS) methyl peaks at 0.00 ppm. The same peak also was used as a chemical shape indicator (ie, as an internal standard for quantitation). Forty-five metabolites were quantified in the 1D ^1^H NMR spectra of serum extracts using the Profiler module of the Chenomx NMR Suite Professional version number 10 software with a built in 1D spectral library. The quantitation was based on comparing the area of selected metabolite peaks with the area under the DSS methyl peak, which corresponded to a known concentration of 0.5 mM in all samples. All metabolite concentrations were expressed in μM/L. The resulting metabolite concentration tables (39 metabolites × 32 samples) were exported to Excel, where sample identifiers were added.

### Statistical analysis

Metabolome data were uploaded to MetaboAnalyst 6.0 and the data were median-normalized and Pareto-scaled before statistical and bioinformatics analyses.^46^ The following comparisons were made: (1) CPM-c vs. EHPM-c at baseline to verify whether the animals started from the same basal metabolic profile; (2) CPM-c vs. EHPM-c after 45 days of consumption of experimental diets to evaluate the effect of the treatments on the serum metabolic profile. Univariate statistical analysis was performed using Student’s *t*-tests, in which differences were considered significant when *P* ≤ .05. Unsupervised (principal component analysis [PCA]) and supervised (orthogonal partial least square discriminant analysis [OPLS-DA]) analyses also were performed. The significances of the group patterns in the PCA were evaluated using PERMANOVA. In the OPLS-DA, variable importance in projection (VIP) was used to rank the metabolites based on their importance in discriminating groups. Based on VIP scores, compounds that contributed most (VIP > 1.0) to the overall model separation and the classification accuracy in the OPLS-DA plot between treatments were highlighted. Moreover, a quantitative enrichment analysis was performed using metabolite quantification datasets for each treatment. Compound names were standardized according to Human Metabolome Database IDs, and the Small Molecule Pathway Database was used as the reference library. Enrichment analysis was based on the global test method,^47^ in which the enrichment ratio represents the proportion of significantly altered metabolites mapped to each metabolic pathway relative to the total number of identified metabolites in the dataset.

## Results

Given the nutritional properties of EHPM-c, our study was designed under the hypothesis that its inclusion in the diet of obese cats could promote specific metabolic adaptations. Specifically, we aimed to investigate whether EHPM-c could modulate key metabolic pathways involved in amino acid and lipid mobilization to affect energy management, thereby supporting its potential application as a nutritional tool in the management of obesity in cats.

Based on 1D 1H NMR analyses, 39 metabolites were identified in serum of CG and TG cats at baseline and after 45 days of consumption of experimental diet, these included essential and non-essential amino acids, metabolites related to glycolysis, citric acid cycle, and purine metabolism; fatty acids; nitrogen; and others (Table S3).

**Figure 1:**
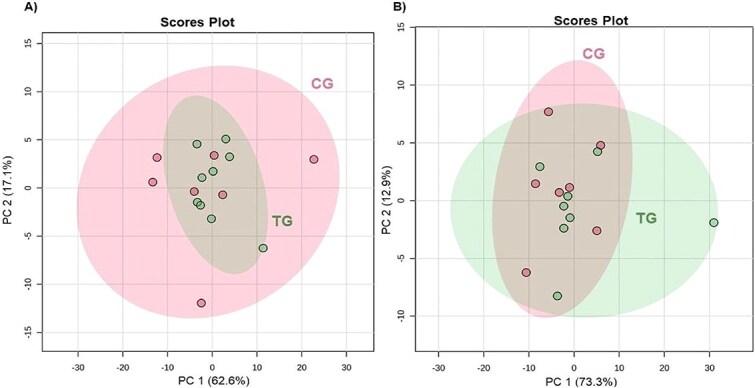
Principal component analysis (PCA) score plot of metabolome distribution. Each circle represents one serum sample from an individual cat. The axes represent principal component 1 (PC1) and principal component 2 (PC2). Colored ellipses indicate the 95% confidence regions (Hotelling’s *t*^2^) for each group. (A) Metabolome distribution across control group (CG) and test group (TG) at time zero (baseline). (B) Metabolome distribution across treatments after 45 days. PCA score plots of serum metabolome distribution. Statistical comparison between CG and TG after 45 days was performed using Student’s *t*-test, and differences were considered statistically significant at *P* ≤ .05.

**Figure 2:**
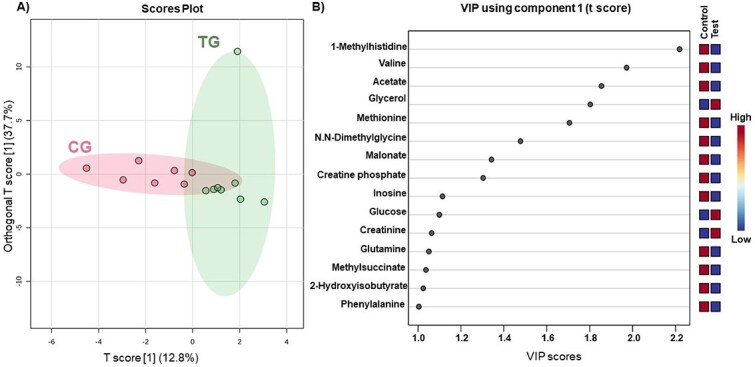
Orthogonal partial least squares discriminant analysis (OPLS-DA) score plot. Each circle represents one serum sample from an individual cat. Colored ellipses indicate the 95% confidence regions (Hotelling’s T^2^) for each group. (A) Metabolome distribution across control group (CG) and test group (TG) at treatments after 45 days. (B) In the variable importance in projection (VIP) score, the colored “control” and “test” columns indicate the relative concentration levels of each metabolite in the control and test groups, respectively, with the color gradient ranging from blue (low concentration) to red (high concentration). The higher the VIP score, the more important the metabolite is in explaining the differences between groups. As a general rule, variables with a VIP score > 1.0 are considered significant contributors to the model.

At baseline (day 0), PCA showed no group segregation (*P* = .87; Figure 1A), indicating similar basal metabolic profiles. Butyrate was the only metabolite differing between groups (CG: 12.0 ± 1.75; TG: 9.3 ± 1.75; *P* = 0.05; Table 2). After 45 days of diet consumption, PCA again showed no separation (*P* = 0.51; Figure 1B). However, compared with controls, the test group had higher glycerol (CG: 185.7 ± 15.21; TG: 211.0 ± 15.21; *P* = .03) and lower valine (91.3 ± 5.07 vs 87.6 ± 5.07; *P* = .01), 1-methylhistidine (17.1 ± 0.61 vs 14.2 ± 0.61; *P* = .01), and acetate (36.9 ± 1.99 vs 31.9 ± 1.99; *P* = .02; Table 3). The OPLS-DA identified distinct clusters between treatments with moderate-to-low predictive ability (*R*^2^ = 0.681; *Q*^2^ = 0.171; Figure 2A). As expected for a supervised method, OPLS-DA emphasized group separation compared with unsupervised PCA. Fifteen metabolites (VIP > 1.0) contributed most to group discrimination (Figure 2B): 1-methylhistidine, valine, acetate, methionine, N,N-dimethylglycine, malonate, creatine phosphate, inosine, glutamine, methylsuccinate, 2-hydroxyisobutyrate, and phenylalanine were higher in the CG, whereas glycerol, glucose, and creatinine were higher in the TG. Supplementary Table S4 presents the list of all metabolites in the VIP analysis.

**Table 2:** Means, standard error of means (SEM) and probability (*P*-value) of the serum metabolites identified (*n* = 45) in cats at baseline (after the diet standardization period and prior to experimental diets consumption).

**Metabolites (μM/L)**	**Group**		**SEM**	***P*-value**
	**Control**	**Test**		
**1-Methylhistidine**	17.7	15.0	1.17	.32
**1.7-Dimethylxanthine**	12.7	9.5	1.29	.44
**2-Hydroxyisobutyrate**	14.9	7.7	2.61	.24
**3-Hydroxyisobutyrate**	17.4	16.9	1.83	.64
**3-Hydroxyisovalerate**	15.9	11.6	1.99	.06
**Acetate**	38.2	34.8	4.68	.84
**Acetone**	4.1	3.9	0.66	.66
**Alanine**	302.0	324.6	40.38	.95
**Butyrate**	12.0	9.3	1.75	.05
**Citrate**	155.1	149.1	12.04	.79
**Creatine**	44.7	54.2	5.43	.56
**Creatine phosphate**	11.8	11.6	1.68	.38
**Creatinine**	64.0	67.5	5.22	.85
**Dimethyl sulfone**	4.1	3.3	0.40	.65
**Formate**	22.4	22.2	2.12	.62
**Glucose**	3641.5	3383.8	404.19	.73
**Glutamine**	297.8	302.4	18.28	.52
**Glycerol**	222.3	265.3	31.05	.16
**Glycine**	210.7	210.5	30.92	.93
**Histidine**	44.9	42.2	2.50	.37
**Inosine**	10.5	9.1	0.86	.10
**Isoleucine**	37.9	37.2	4.19	.49
**Lactate**	1901.6	1799.9	231.21	.68
**Leucine**	62.8	62.1	5.23	.12
**Lysine**	53.1	57.3	7.07	.92
**Malonate**	29.5	23.2	2.74	.30
**Methionine**	55.0	45.2	6.14	.26
**Methylsuccinate**	19.2	17.5	1.88	.32
**N.N-Dimethylglycine**	2.1	2.0	0.22	.87
**O-Acetylcarnitine**	15.4	12.4	1.99	.76
**Phenylalanine**	49.2	48.9	3.85	.74
**Pyroglutamate**	24.7	24.2	2.48	.87
**Pyruvate**	32.9	34.4	4.25	.70
**Sarcosine**	4.7	5.6	1.06	.76
**Serine**	101.8	100.1	13.51	.75
**Threonine**	80.0	90.6	8.14	.73
**trans-4-Hydroxy-L-proline**	20.3	20.0	1.63	.99
**Tyrosine**	33.6	33.9	3.15	.79
**Valine**	100.3	104.3	9.34	.94

Metabolite concentrations (μM/L) in serum of cats fed control (CPM-c) or experimental (EHPM-c) diets. Univariate statistical analysis was performed using Student’s *t*-test (*P* ≤ .05).

**Table 3:** Abundance of main serum metabolites identified after 45 days of consumption of the control and test experimental diets.

**Metabolites (μM/L)**	**Group**		**SEM**	***P*-value**
	**Control**	**Test**		
**1-Methylhistidine**	17.1	14.2	0.61	.01
**1.7-Dimethylxanthine**	8.6	7.9	0.71	.93
**2-Hydroxyisobutyrate**	11.6	9.7	2.47	.32
**3-Hydroxyisobutyrate**	13.7	15.1	0.77	.91
**3-Hydroxyisovalerate**	12.7	8.4	1.51	.18
**Acetate**	36.9	31.9	1.99	.02
**Acetone**	3.3	3.2	0.27	.94
**Alanine**	257.8	312.0	21.65	.49
**Butyrate**	7.7	7.9	0.92	.42
**Citrate**	145.0	154.9	9.24	.85
**Creatine**	44.8	47.6	4.51	.84
**Creatine phosphate**	11.7	10.8	0.71	.18
**Creatinine**	57.3	62.0	3.76	.33
**Dimethyl sulfone**	4.4	4.5	0.42	.93
**Formate**	21.4	19.0	1.08	.48
**Glucose**	2809.2	2664.7	174.85	.30
**Glutamine**	350.4	346.4	14.07	.85
**Glycerol**	185.7	211.0	15.21	.03
**Glycine**	155.8	155.2	10.38	.16
**Histidine**	46.2	46.4	2.07	.44
**Inosine**	9.4	7.3	0.56	.50
**Isoleucine**	34.2	30.5	2.40	.57
**Lactate**	1490.0	1550.4	97.99	.83
**Leucine**	59.2	57.4	3.29	.89
**Lysine**	55.4	55.4	3.01	.90
**Malonate**	27.0	24.6	1.49	.20
**Methionine**	49.6	45.0	2.98	.09
**Methylsuccinate**	14.7	14.5	0.67	.46
**N.N-Dimethylglycine**	1.9	2.1	0.13	.25
**O-Acetylcarnitine**	12.6	10.5	0.59	.82
**Phenylalanine**	49.0	49.0	2.36	.43
**Pyroglutamate**	24.0	24.3	2.42	.85
**Pyruvate**	33.9	33.1	4.10	.56
**Sarcosine**	3.8	4.3	0.68	.56
**Serine**	92.7	103.7	5.71	.61
**Threonine**	73.7	76.0	4.33	.65
**trans-4-Hydroxy-L-proline**	21.2	23.0	2.10	.33
**Tyrosine**	26.4	31.9	1.90	.88
**Valine**	91.3	87.6	5.07	.01

Metabolite concentrations (μM/L) in serum of cats fed control (CPM-c) or experimental (EHPM-c) diets. Univariate statistical analysis was performed using Student’s *t*-test (*P* ≤ .05).

**Figure 3:**
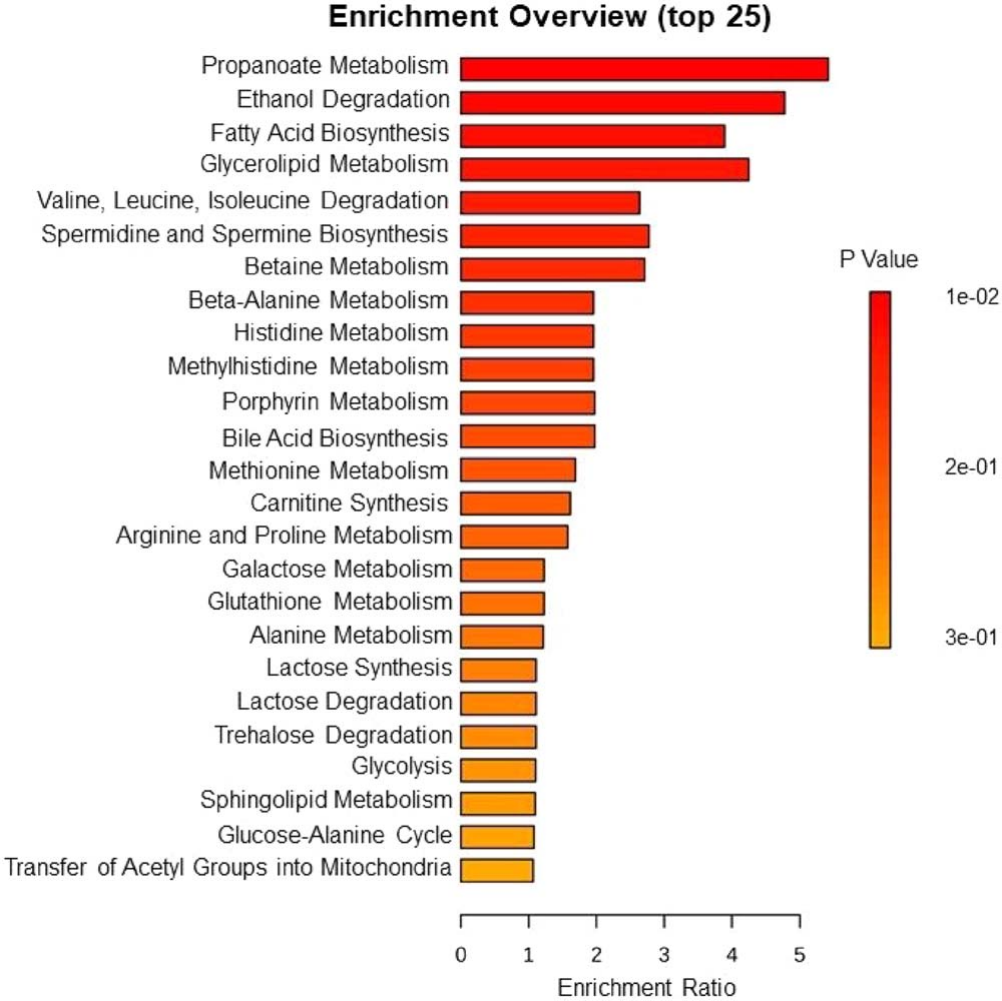
Top 25 enriched pathways identified through quantitative enrichment analysis of the serum metabolites (*n* = 45) in cats after 45 days of experimental diet consumption. The enrichment ratio represents the proportion of identified metabolites associated with each pathway relative to the total number of metabolites detected in the dataset. The color gradient indicates the adjusted *P*-value (*P* ≤ .10) for each enriched pathway.

Quantitative enrichment analysis (Figure 3 and Table 4) determined that the main metabolic pathways affected by the treatments after 45 days of experimental diets were correlated with energy metabolism, which included propanoate metabolism (*P* = .01), ethanol degradation (*P* = .02), fatty acid biosynthesis (*P* = .02), and glycerolipid metabolism (*P* = .03).

**Table 4:** Description of top 25 enriched terms in the quantitative enrichment analysis of the serum metabolites identified and quantified (*n* = 45) in cats after 45 days of experimental diets consumption.

**Pathway name**	**Total**	**Hits**	***P*-value**	**Matched compound**			
**Propanoate metabolism**	42	1	0.01	Valine			
**Ethanol degradation**	19	1	0.02	Acetic acid			
**Fatty acid biosynthesis**	35	2	0.02	Butyric acid	Acetic acid		
**Glycerolipid metabolism**	25	1	0.03	Glycerol			
**Valine, leucine and isoleucine degradation**	59	4	0.05	3-Hydroxyisobutyric acid	Isoleucine	Leucine	Valine
**Spermidine and spermine biosynthesis**	18	1	0.09	Methionine			
**Betaine metabolism**	21	2	0.09	Dimethylglycine	Methionine		
**β-Alanine metabolism**	34	2	0.14				
**Histidine metabolism**	42	2	0.14				
**Methylhistidine metabolism**	4	2	0.14				
**Porphyrin metabolism**	40	1	0.16				
**Bile acid biosynthesis**	65	1	0.16				
**Methionine metabolism**	42	5	0.17				
**Carnitine synthesis**	22	2	0.20				
**Arginine and proline metabolism**	52	4	0.20				
**Galactose metabolism**	38	2	0.28				
**Glutathione metabolism**	20	3	0.29				
**Alanine metabolism**	17	3	0.29				
**Lactose synthesis**	19	1	0.30				
**Lactose degradation**	9	1	0.30				
**Trehalose degradation**	11	1	0.30				
**Glycolysis**	23	2	0.31				
**Sphingolipid metabolism**	40	2	0.31				
**Glucose-alanine cycle**	13	3	0.31				
**Transfer of acetyl groups into mitochondria**	22	3	0.31				

Quantitative enrichment analysis was conducted using the Small Molecule Pathway Database (SMPDB) libraries, with compound names standardized according to the Human Metabolome Database (HMDB) identifiers. Statistical significance of group patterns was assessed using PERMANOVA; pathways with *P* ≤ .10 were considered enriched.

## Discussion

Our results suggest that incorporating EHPM-c into the diet of obese cats can modify serum metabolomic profiles. The observed changes in metabolite concentrations were linked to alterations in metabolic pathway activity. The metabolomic differences were significant and biologically relevant, because the altered metabolites and enriched pathways reflect meaningful metabolic adaptations in obese cats.

Specifically, valine, 1-methylhistidine, and acetate decreased, whereas glycerol increased in the test group compared with controls. These changes coincided with enrichment of propanoate metabolism, ethanol degradation, fatty acid biosynthesis, and glycerolipid metabolism, likely indicating enhanced amino acid modulation and lipid mobilization.^15^^,^^32^^,^^33^

These findings suggest EHPM-c may optimize energy metabolism by promoting lipolysis and modulating amino acid degradation. Overall, EHPM-c emerges as a promising nutritional strategy for managing obesity in cats. However, controlled caloric restriction remains the primary evidence-based approach, and the ability of EHPM-c to enhance weight loss has not yet been demonstrated. The observed metabolic modulation may support weight loss programs by preserving lean body mass and improving energy metabolism, but additional studies are needed to evaluate its effectiveness in calorie-restricted diets.

Branched chain amino acids, such as leucine, isoleucine, and valine, play a key anabolic role in muscle protein synthesis,^48^^,^^49^ mainly via activation of the mammalian target of rapamycin pathway.^50^ Conversely, BCAA catabolism is increasingly linked to metabolic dysfunction, contributing to obesity and type 2 diabetes in humans.^51^ Several studies show that increased plasma BCAA concentrations correlate with insulin resistance and glucose intolerance.^52–57^

As in humans, obesity in cats can promote type 2 diabetes, characterized by insulin resistance. However, the role of BCAAs in disease metabolism in cats remains poorly understood. Previous studies found no differences in serum BCAA concentrations in obese cats before and after weight loss.^14^^,^^15^ Furthermore, one of these studies observed no variations among obese, obese-reduced, and lean cats.^15^ In contrast, another study reported decrease leucine and valine in cats with ideal BCS in diabetic remission but could not link this finding to body composition.^39^ In our study, only valine decreased in the EHPM-c diet group, indicating preliminary positive modulation of energy metabolism in obese cats. This decrease in valine is especially notable together with the enrichment of propanoate metabolism, because valine was the metabolite driving this pathway’s regulation.

Propanoate metabolism is a key biochemical pathway processing propionyl-CoA, an intermediate from catabolism of certain BCAAs such as valine and oxidation of some branched chain fatty acids.^58^ It acts as a metabolic crossroad, connecting these catabolic processes to core energy pathways, including the tricarboxylic acid (TCA) cycle and gluconeogenesis.^59^^,^^60^

After initial steps common to BCAAs, valine undergoes reactions yielding propionyl-CoA as a major end product. The increased availability of this substrate likely drives the enrichment of propanoate metabolism observed, highlighting a substrate-driven regulatory mechanism.^61^ The conversion of propionyl-CoA into succinyl-CoA is especially relevant, because succinyl-CoA feeds directly into the TCA cycle, supporting energy production and biosynthetic demands.^60^^,^^61^

Beyond the TCA cycle, propanoate metabolism also contributes to gluconeogenesis, because propionate is an important non-carbohydrate glucose precursor.^62^ This factor suggests that enhanced valine catabolism, via increased propionyl-CoA, supports TCA flux and glucose regulation, potentially beneficial for metabolic management of obesity in cats.

In obese humans under caloric restriction, high BCAA and catabolite concentrations are linked to increased protein degradation, releasing BCAAs to meet energy demands, which can lead to lean mass loss.^63^ Thus, the decrease in BCAAs in our study may reflect lower protein degradation for energy, indicating a favorable metabolic effect for obese cats.^57^ Moreover, because accumulation of these metabolites may contribute to type 2 diabetes in cats, their reduction could have clinical relevance for disease prevention.

The modulation of this pathway in obese cats fed the test diet may reflect a metabolic shift toward increased fatty acid oxidation and use of alternative substrates for adenosine triphosphate synthesis, beneficial for obese cats under weight loss and caloric restriction. As obligate carnivores, domestic cats rely heavily on gluconeogenesis,^64^ and enhanced propanoate metabolism may spare dietary and endogenous amino acids from gluconeogenic use.^65^^,^^66^ This modulation suggests increased lipid mobilization and metabolic adaptability, potentially supporting lean mass preservation during EHPM-c dietary intervention.

Preserving muscle mass is a critical metabolic priority for strict carnivores, whose amino acid metabolism sustains energy balance and protein turnover.^67^ Among these, histidine is essential not only for protein synthesis but also for glycogen metabolism.^44^^,^^68^ Its catabolite, 1-methylhistidine, serves as a biomarker of muscle protein breakdown,^68^ providing insights into muscle preservation under caloric restriction.

Previous studies have emphasized the relevance of 1-methylhistidine and related metabolites in chronic diseases. In dogs with congestive heart failure (CHF), methylhistidine and histidine pathways were overrepresented, with advanced-stage animals (C and D) showing increased 1-methylhistidine and 3-methylhistidine compared with less affected groups.^69^ Moreover, in cachexia linked to chronic diseases, a previous study observed significant increases in 1-methylhistidine and N-acetyl-1-methylhistidine when comparing patients with CHF cachexia those without cachexia and lower severity groups.^70^

With regard to cats, the link between 1-methylhistidine and energy metabolism remains poorly understood. A previous study examined the role of choline in overweight male cats using a dose–response model but found no significant changes in serum 1-methylhistidine concentrations with different doses.^36^ Conversely, another study showed that increased concentrations of this metabolite strongly predicted long-term weight loss in obese cats,^14^ reinforcing its value as a metabolic biomarker.

Thus, the decreased serum 1-methylhistidine observed in our study suggests lower protein turnover and muscle degradation, effects relevant for preserving lean mass during weight loss. The inclusion of EHPM-c emerges as a promising dietary strategy for obese cats, because its ability to enhance dietary protein utilization may help maintain or increase muscle mass under caloric restriction, limiting protein loss, and preventing cachexia.

Beyond its effects on protein metabolism, another crucial aspect influencing metabolic health in obese cats involves energy substrates such as acetate, which plays a role in modulating energy and lipid metabolism. Acetate is a short-chain fatty acid with a central role in energy metabolism, particularly in conditions associated with metabolic dysfunction, such as obesity and type 2 diabetes.^71^ Its relevance stems from its function as a metabolic intermediate that can be readily converted into acetyl coenzyme A (acetyl-CoA), a key molecule in energy production in the TCA^72^ and in lipogenesis.^73^^,^^74^ Moreover, acetate exerts regulatory functions by modulating the oxidative capacity of tissues through protein kinase phosphorylation.^75^ In humans, increased circulating acetate has been associated with enhanced fat oxidation, increased energy expenditure,^76^ and improved insulin sensitivity, along with decreased insulin resistance.^77^^,^^78^

In cats, increased acetoacetate, a downstream product of acetate, has been reported in obese cats under caloric restriction, indicating enhanced lipid oxidation.^14^^,^^15^ However, a previous study found no significant differences in acetoacetate concentrations between lean and energy-restricted obese cats, questioning the consistency of this response.^15^ In our study, without caloric restriction, acetate concentrations decreased in the test group, suggesting that acetate dynamics depend on both energy restriction and diet-induced metabolic states. Increased fatty acid oxidation increases acetyl-CoA production, which may decrease amino acid catabolism for energy, limiting TCA intermediates such as oxaloacetate^79^ and consequently decreasing acetoacetate and acetate production.

A related pathway involves ethanol metabolism, a known source of circulating acetate, mainly hepatic, through ethanol conversion to acetaldehyde and then acetate.^80^ Once formed, acetate enters circulation, reaching peripheral tissues,^73^^,^^74^ acting as a metabolic bridge that channels carbon from ethanol into lipogenesis. Acetate is rapidly converted to acetyl-CoA by acetyl-coenzyme A synthetase,^73^ fueling de novo lipogenesis.^81^

Our results showed enrichment of ethanol degradation pathways in the TG, indicating a shift in energy metabolism. Endogenous ethanol production in mammals originates largely from gut microbiota fermentation, involving taxa such as Proteobacteria (Enterobacteriaceae) and Firmicutes (Clostridiaceae, Lactobacillaceae).^82^ The hydrolyzed protein in our test diet supplied peptides preferentially fermented by intestinal bacteria, unlike free amino acids. Although no significant α- or β-diversity differences were detected between diets,^28^ time-dependent modulations occurred in ethanol-producing families like Enterobacteriaceae and Clostridiaceae.^28^ This finding suggests that whereas microbial composition remained stable, dietary intervention altered functional metabolic outputs, increasing endogenous ethanol production and subsequent ethanol degradation.

It is plausible that the metabolic shifts observed were partly mediated by gut microbiota activity. Previous research shows that obesity in cats alters gut microbial composition and metabolic output, including the Firmicutes/Bacteroidetes ratio and short-chain fatty acid production.^83–85^ Such microbial changes modulate host lipid and amino acid metabolism through metabolites such as acetate, propionate, and butyrate.^86^^,^^87^ Therefore, enrichment of propionate metabolism, ethanol degradation, and glycerolipid metabolism may reflect both host metabolic adaptations and microbial fermentation of dietary substrates from the EHPM-c. These host microbiota interactions likely contribute to lipid mobilization and amino acid catabolism regulation, supporting that EHPM-c influences systemic metabolism via nutritional and microbial mechanisms.

Interestingly, despite ethanol degradation pathway enrichment, circulating acetate decreased in the TG. In the absence of prior reports, this finding may indicate increased acetate oxidation for energy. This paradox likely reflects enhanced lipid utilization efficiency in obese cats, decreasing reliance on gluconeogenic amino acids as lipid-derived energy becomes predominant. The enrichment of ethanol degradation and fatty acid biosynthesis pathways supports this view, because both converge on acetyl-CoA, a key precursor for lipogenesis via acetyl-CoA synthetase and carboxylase activity.^88^

In the TG, fatty acid biosynthesis also was significantly enriched. Although seemingly contradictory, this finding likely represents a compensatory mechanism.^89^^,^^90^ Because lipid oxidation increases to meet energy demands, lipogenesis may act to preserve lipid homeostasis, preventing excessive depletion.^90^ This adaptive response balances lipid degradation and synthesis, ensuring metabolic stability amid substrate utilization changes.^91^ The decrease in acetate, increase in glycerol, and activation of propanoate, ethanol degradation, and glycerolipid metabolism pathways further support this regulated metabolic compensation.

Clinically, such metabolic flexibility has major implications. The decrease in circulating acetate alongside enhanced ethanol degradation suggests correction of a dysregulated metabolic state.^92^ If the CG reflects obesity-related metabolic inflexibility, increased acetate could serve as a marker of this dysfunction. Conversely, the decreased acetate in the TG likely indicates metabolic normalization, with improved substrate utilization and lower metabolic stress.

This adaptive capacity is particularly important for managing obesity in cats. Controlled modulation of lipid metabolism, balancing oxidation and biosynthesis, may decrease the risk of hepatic lipidosis, a potentially fatal outcome of rapid fat mobilization.^93^ Moreover, systemic acetate modulation may reflect metabolic health and serve as a therapeutic target.

Although acetate dynamics offer insight into lipid and metabolic regulation, other markers of energy balance, such as glycerol, also are critical. Visceral fat lipolysis releases free fatty acids, driving lipid oxidation,^15^ whereas glycerol binds fatty acids to form triglycerides, stored in adipose tissue and released during lipolysis. In humans, caloric restriction increases serum glycerol concentrations, indicating increased lipolysis.^63^ Similarly, a previous study found higher glycerol in obese cats during weight loss, reflecting enhanced lipid mobilization.^14^ Our study also identified increased glycerol concentrations associated with lipolysis.

These findings suggest that increased glycerol reflects intensified lipolysis and decreased triglyceride synthesis, both beneficial metabolic adaptations in obesity. Notably, all cats remained obese throughout the trial, with no significant weight loss or BCS differences between groups. This metabolic responsiveness despite stable phenotype suggests that prolonged dietary administration may be needed to fully express the diet’s potential in fat mass reduction and obesity management.

When interpreted alongside the other results, the objective is not to validate EHPM-c as a lipolytic ingredient, but to assess its ability to modulate energy metabolism in obese cats without caloric restriction. Its dietary inclusion may promote amino acid preservation, decreasing their oxidation for energy while stimulating triglyceride mobilization from adipose tissue to meet energy demands. Because amino acid and lipid metabolism are inherently dysregulated in obesity,^5^ the higher activation of glycerolipid metabolism in the test group further supports enhanced lipid mobilization and increased fat utilization through lipolysis.

A parallel can be drawn with hepatic lipidosis in cats, a condition in which negative energy balance in obese cats disrupts fatty acid uptake, synthesis, degradation, and secretion.^93^ In this regard, previous studies have identified several metabolic pathways altered in hepatic lipidosis, including glycerolipid metabolism.^94^^,^^95^ The overactivation of this pathway suggests an imbalance between lipid synthesis and degradation, potentially contributing to disease progression.

From a metabolic perspective, a previous study reported that obese cats exhibit more fatty acid release, predominant lipid oxidation, and increased lipolytic activity compared with lean cats, which preferentially oxidize glucose.^95^ However, these differences were observed between groups differing in body composition and without energy restriction. Similarly, another study found that, although caloric restriction did not significantly alter glycerol concentrations, they remained increased in obese cats without restriction, suggesting higher circulating fatty acids and enhanced lipolysis.^15^

In our study, all cats were obese and maintained identical body composition and caloric intake. The only distinguishing factor was the inclusion of EHPM-c, which was associated with increased glycerolipid metabolism activation and increased serum glycerol concentrations, indicating enhanced lipolysis and lipid utilization. This finding likely resulted in more circulating fatty acid availability and increased reliance on fat as an energy substrate.

Collectively, these results emphasize the potential of targeted nutritional interventions to modulate amino acid and lipid metabolism in obese cats. The combination of increased 1-methylhistidine degradation and enhanced lipid mobilization suggests that EHPM-c may improve metabolic efficiency while preserving muscle mass. Moreover, the observed increase in BCAA degradation could contribute to improved insulin sensitivity, as reported in humans, thereby supporting weight management and mitigating obesity-related comorbidities, including type 2 diabetes.

Our findings should be interpreted in light of the limitations of our study. The adopted approach provides novel insights into the metabolic effects of EHPM-c inclusion in obese cats. To ensure standardization, cats were exclusively fed the experimental diets without additional treats or snacks, which may have influenced overall nutrient intake. Nevertheless, the absence of significant baseline differences in variables such as sex, age, body weight, BCS, season, breed, and health or welfare indicators indicates successful randomization and minimizes potential bias.

Other limitations include the small sample size, short duration, and the use of privately owned cats. Additionally, thyroid hormone concentrations were not measured, which prevents definitive exclusion of hyperthyroidism. Although no enrolled cat showed clinical signs typically associated with hyperthyroidism, (eg, weight loss, behavioral changes), the absence of overt clinical signs does not confirm a euthyroid state. The use of crude fiber analysis instead of total dietary fiber is another limitation, because it may underestimate the true fiber content and affect the interpretation of fiber-related metabolic outcomes. A larger sample would have enhanced statistical power and improved the generalizability of the results. Although the 45-day period was sufficient to detect early metabolic responses, it may not have captured long-term adaptations. Finally, maintaining cats in household environments introduces variability in stress, lifestyle, and compliance with the feeding protocol, which, despite standardization and randomization, may have acted as a confounding factor.

## Conclusions

The inclusion of EHPM-c in the diet of obese cats promoted beneficial metabolic adaptations, suggesting an optimization of energy metabolism. The observed increase in lipid mobilization and modulation of amino acid metabolism suggests changes toward enhanced fat utilization, while potentially preserving muscle mass. These findings highlight the potential use of EHPM-c as a functional ingredient in diets aimed at treating obesity in cats and facilitating weight loss programs.

## Supplementary Material

aalaf075_SupplementaryTables

## Off-label antimicrobial declaration

Authors declare no off-label use of antimicrobials.

## Institutional animal care and use committee or other approval declaration

The animal studies were approved by the Ethic Committee on Animal Use of the School of Veterinary Medicine and Animal Science at the University of São Paulo (CEUA/FMVZ). The study was approved under protocol number 8609280422. The studies were conducted in accordance with the local legislation and institutional requirements. Written informed consent was obtained from the owners for the participation of their animals in this study.

## Abbreviations

1D: one-dimensional
AceCS: acetyl-coenzyme A synthetase
ALT: alanine aminotransferase
AST: aspartate aminotransferase
ATP: adenosine triphosphate
BCAAs: branched-chain amino acids
BCS: body condition score
BHA: butylated hydroxyanisole
BHT: butylated hydroxytoluene
CG: control group
CHF: congestive heart failure
CPM-c: conventional poultry byproduct meal
DSS: 2,2-dimethyl-2-silapentane-5-sulfonate
EHPM-c: enzymatically hydrolyzed poultry byproduct meal
FHL: feline hepatic liposis
HMDB: Human Metabolome Database
IKW: isoleucine-lysine-tryptophan
LKA: leucine-lysine-alanine
LKP: leucine-lysine-proline
NMR: nuclear magnetic resonance
OPLS-DA: orthogonal partial least squares discriminant analysis
PCA: principal component analysis
SCFAs: short-chain fatty acids
SMPDB: Small Molecule Pathway Database
T0: 0 day
T45: 45 days
TCA: tricarboxylic acid
TG: test group
VIP: variable importance in the projection
YYRA: tyrosine-tyrosine-arginine-alanine
