# Supplementary material for: Serum metabolomics identifies metabolic changes in obese cats fed enzymatically hydrolyzed poultry byproduct meal
Source: J Vet Intern Med. 2026 Jan 21;40(1):aalaf075. doi: 10.1093/jvimsj/aalaf075 (PMC12881970; doi:10.1093/jvimsj/aalaf075)
Supplement: aalaf075_SupplementaryTables [file aalaf075_supplementarytables.docx]

| **Supplementary Table 1**. Individual characteristics of cats from control (CPM-c) and test (EHPM-c) groups, including age, breed, gender, neuter status, metabolizable energy requirement (MER), initial and final body weight (BW), body condition score (BCS), muscle mass score (MMS), and systolic blood pressure (SBP) | | | | | | | | | | | | | | | |
| --- | --- | --- | --- | --- | --- | --- | --- | --- | --- | --- | --- | --- | --- | --- | --- |
| **Animal Number** | **Treatment** | **Age (years)** | **Breed** | **Gender** | **Neuter status** | **MER (kcal)** | **Initial** | | |  | **Final** | | |  | |
|  |  |  |  |  |  |  | **BW (kg)** | **BCS** | **MMS** | **SBP (mmHg)** | **BW (kg)** | **BCS** | **MMS** | | **SBP (mmHg)** |
| 1 | Control (CPM-c) | 7 | Mixed-breed | Male | Neutered | 245.483 | 4.9 | 8 | 2 | 140 | 4.9 | 8 | 2 | | 140 |
| 2 | Test (EHPM-c) | 7 | Mixed-breed | Male | Neutered | 279.864 | 6.8 | 8 | 2 | 125 | 6.8 | 8 | 2 | | 140 |
| 3 | Test (EHPM-c) | 9 | Mixed-breed | Female | Neutered | 247.475 | 5.0 | 8 | 2 | 140 | 5.0 | 8 | 2 | | 130 |
| 4 | Test (EHPM-c) | 7 | Mixed-breed | Male | Neutered | 279.864 | 6.8 | 9 | 3 | 135 | 6.9 | 9 | 3 | | 135 |
| 5 | Control (CPM-c) | 10 | Mixed-breed | Female | Neutered | 269.712 | 6.2 | 9 | 3 | 130 | 6.3 | 9 | 3 | | 145 |
| 6 | Control (CPM-c) | 7 | Mixed-breed | Male | Neutered | 262.612 | 5.8 | 9 | 3 | 145 | 6.1 | 9 | 3 | | 115 |
| 7 | Control (CPM-c) | 7 | Mixed-breed | Male | Neutered | 241.425 | 4.7 | 8 | 3 | 140 | 5.2 | 8 | 3 | | 140 |
| 8 | Test (EHPM-c) | 12 | Mixed-breed | Female | Neutered | 271.444 | 6.3 | 9 | 3 | 130 | 6.7 | 9 | 3 | | 120 |
| 9 | Test (EHPM-c) | 8 | Mixed-breed | Male | Neutered | 269.712 | 6.2 | 9 | 3 | 140 | 6.6 | 9 | 3 | | 160 |
| 10 | Control (CPM-c) | 7 | Mixed-breed | Male | Neutered | 257.092 | 5.5 | 9 | 3 | 130 | 5.5 | 9 | 3 | | 150 |
| 11 | Control (CPM-c) | 9 | Mixed-breed | Male | Neutered | 269.712 | 6.2 | 9 | 3 | 125 | 6.6 | 9 | 3 | | 140 |
| 12 | Test (EHPM-c) | 7 | Mixed-breed | Male | Neutered | 253.311 | 5.3 | 8 | 2 | 95 | 5.7 | 8 | 2 | | 120 |
| 13 | Test (EHPM-c) | 7 | Mixed-breed | Female | Neutered | 251.388 | 5.2 | 8 | 3 | 130 | 5.2 | 8 | 3 | | 130 |
| 14 | Control (CPM-c) | 12 | Mixed-breed | Male | Neutered | 287.92 | 7.3 | 9 | 2 | 115 | 7.1 | 9 | 2 | | 130 |
| 15 | Test (EHPM-c) | 7 | Mixed-breed | Male | Neutered | 276.542 | 6.6 | 8 | 3 | 140 | 6.4 | 8 | 3 | | 120 |
| 16 | Test (EHPM-c) | 7 | Mixed-breed | Male | Neutered | 274.858 | 7.0 | 9 | 3 | 140 | 6.5 | 9 | 3 | | 125 |
| Legend: Conventional poultry byproduct meal (CPM-c); Hydrolyzed poultry byproduct meal (EHPM-c). Classification of systemic blood pressure and associated risk of target organ damage in cats. Blood pressure categories follow the ACVIM consensus statement for the identification, evaluation, and management of systemic hypertension in dogs and cats (Acierno et al., 2020). Cats are classified as normotensive (<140 mmHg, minimal risk), pre-hypertensive (140–159 mmHg, low risk), hypertensive (160–179 mmHg, moderate risk), or severely hypertensive (>180 mmHg, high risk). | | | | | | | | | | | | | | | |

| **Supplementary Table 2**. Individual baseline laboratory parameters used for health screening of cats | | | | | | | | | | | | |
| --- | --- | --- | --- | --- | --- | --- | --- | --- | --- | --- | --- | --- |
| **Animal Number** | **Urea (mg/dL)** | **Creatine (mg/dL)** | **ALT (UI/L)** | **AST (UI/L)** | **Alkaline phosphatase (UI/L)** | **GGT (UI/L)** | **Total protein (g/dL)** | **Albumin (g/dL)** | **Globulins (g/dL)** | **Cholesterol (mg/dL)** | **Triglycerides (mg/dL)** | **Glucose (mg/dL)** |
|  |  |  |  |  |  |  |  |  |  |  |  |  |
| 1 | 44.2 | 1.2 | 38.0 | 38.0 | 51.0 | 1.0 | 7.1 | 3.1 | 4.4 | 154.1 | 23.9 | 93.1 |
| 2 | 68.3 | 1.4 | 90.0 | 28.0 | 23.0 | 1.0 | 6.8 | 3.3 | 3.4 | 175.3 | 64.7 | 73.0 |
| 3 | 47.4 | 1.3 | 45.0 | 20.0 | 21.0 | 0.0 | 7.3 | 3.0 | 4.2 | 161.3 | 32.8 | 103.7 |
| 4 | 53.3 | 1.6 | 63.0 | 30.0 | 42.0 | 0.0 | 7.5 | 3.5 | 4.2 | 167.3 | 30.4 | 88.9 |
| 5 | 47.6 | 1.4 | 67.0 | 36.0 | 37.0 | 0.0 | 6.8 | 3.5 | 3.2 | 176.6 | 32.1 | 96.4 |
| 6 | 55.5 | 1.6 | 70.0 | 25.0 | 30.0 | 1.0 | 7.4 | 3.2 | 4.4 | 44.0 | 48.0 | 100.2 |
| 7 | 44.4 | 1.5 | 49.0 | 26.0 | 38.0 | 0.0 | 7.2 | 3.4 | 3.7 | 34.5 | 91.2 | 86.0 |
| 8 | 56.7 | 1.5 | 60.0 | 35.0 | 45.0 | 0.0 | 7.7 | 2.8 | 3.9 | 48.9 | 95.2 | 94.0 |
| 9 | 50.9 | 1.5 | 60.0 | 44.0 | 35.0 | 1.0 | 6.6 | 3.0 | 2.7 | 49.4 | 90.7 | 81.0 |
| 10 | 41.9 | 1.6 | 59.0 | 30.0 | 44.0 | 0.0 | 6.4 | 3.1 | 3.0 | 154.1 | 23.9 | 93.1 |
| 11 | 52.0 | 1.6 | 54.0 | 32.0 | 43.0 | 1.0 | 7.4 | 3.3 | 3.9 | 54.4 | 64.7 | 73.0 |
| 12 | 46.4 | 1.6 | 75.0 | 37.0 | 52.0 | 1.0 | 7.0 | 2.5 | 2.9 | 161.3 | 32.8 | 103.7 |
| 13 | 45.4 | 1.3 | 65.0 | 31.0 | 26.0 | 0.0 | 7.5 | 3.4 | 4.0 | 167.3 | 30.4 | 88.9 |
| 14 | 50.8 | 1.7 | 60.0 | 46.0 | 37.0 | 0.0 | 6.9 | 3.2 | 3.5 | 163.2 | 37.0 | 139.7 |
| 15 | 48.6 | 1.4 | 43.0 | 26.0 | 37.0 | 1.0 | 8.2 | 3.0 | 4.2 | 139.3 | 29.4 | 101.2 |
| 16 | 55.4 | 1.4 | 92.0 | 32.0 | 23.0 | 0.0 | 7.1 | 3.1 | 4.0 | 97.6 | 39.6 | 74.0 |
| **Reference values*** | 38.6 - 70.7 | 0.7 - 1.8 | 30.0 - 100 | 12.0 - 56.0 | 15.0 - 92.0 | 0.0 - 2.0 | 5.7 - 7.9 | 2.3 - 3.4 | 2.6 - 4.5 | 38.0 - 186.0 | 10.0 - 114.0 | 58.0 - 120.0 |
| Legend: Alanine aminotransferase (ALT); Aspartate aminotransferase (AST); Gamma-glutamyl transferase (GGT).  * Veterinary Laboratory Medicine: Interpretation and Diagnosis. MEYER, D.J.; HARVEY, J.W. Ed. Saunders. 2 ed. 2004. | | | | | | | | | | |  |  |

| **Supplementary Table 3.** Comparison of serum ¹H NMR metabolite concentrations between control and test groups of obese cats at baseline (T0) and day 45 (T45) | | | | | | | | | | | | | | | | | | | | | | | | | | | | | | | | | | |
| --- | --- | --- | --- | --- | --- | --- | --- | --- | --- | --- | --- | --- | --- | --- | --- | --- | --- | --- | --- | --- | --- | --- | --- | --- | --- | --- | --- | --- | --- | --- | --- | --- | --- | --- |
| **Metabolites (μM/L)** | **Baseline (Time 0)** | | | | | | | | | | | | | | | | | **After 45 days (T45)** | | | | | | | | | | | | | | | | |
|  | **Control (CPM-c)** | | | | | | | **Test (EHPM-c)** | | | | | | | | | | **Control (CPM-c)** | | | | | | | | **Test (EHPM-c)** | | | | | | | | |
| **1.7-Dimethylxanthine** | 8.1 | 13.8 | 7 | 8.1 | 23.4 | 10.8 | 17.8 | 4.8 | 6.7 | 7.8 | 9.3 | 7.8 | 7.7 | 14.6 | 8.8 | 17.9 | 6.9 | | 12.9 | 6.8 | 13.5 | 3.5 | 7.4 | 9.3 | 1.3 | | 9.5 | 4.6 | 7.7 | 7.1 | 5.6 | 10.5 | 9.3 | 8.5 |
| **2-Hydroxyisobutyrate** | 10.5 | 6.9 | 23.6 | 34.6 | 23.6 | 3.3 | 2 | 6.5 | 10.7 | 27.7 | 4.2 | 2.4 | 3.7 | 3.3 | 3.1 | 7.7 | 6.1 | | 4.7 | 21.4 | 31.9 | 6.8 | 6.8 | 3.8 | 1.5 | | 18 | 28.8 | 4.9 | 4 | 4.5 | 7.6 | 6.5 | 3.4 |
| **3-Hydroxyisobutyrate** | 19.6 | 17.3 | 15.5 | 12 | 33.7 | 9.7 | 14.3 | 21.7 | 17.1 | 11.7 | 11.1 | 12.9 | 17.4 | 9.4 | 17.6 | 33.5 | 12.5 | | 12.7 | 11.9 | 16.3 | 13.6 | 15.1 | 13.7 | 2.6 | | 19.8 | 13.4 | 20.1 | 16.5 | 12 | 17.5 | 10.8 | 11 |
| **3-Hydroxyisovalerate** | 16.9 | 15.1 | 11.3 | 11.4 | 39.9 | 7.3 | 9.4 | 10.8 | 11.7 | 5.9 | 9.8 | 12.6 | 14.3 | 8.7 | 9.8 | 20.7 | 23.8 | | 12.4 | 13.2 | 21.6 | 4.7 | 7.4 | 5.7 | 4.3 | | 7.9 | 8.7 | 12.4 | 12.7 | 7.8 | 8.7 | 4.2 | 4.5 |
| **Acetate** | 41.9 | 29.9 | 30.4 | 33 | 75.7 | 16.4 | 40.3 | 15.7 | 26.9 | 25 | 19.4 | 32.9 | 43.9 | 41.7 | 26.2 | 81.5 | 45.5 | | 41.1 | 38.6 | 46.9 | 31.7 | 23.6 | 31.1 | 17.3 | | 26 | 32.7 | 47.2 | 28.3 | 30.5 | 32.3 | 27.9 | 30.3 |
| **Acetone** | 2.2 | 4.9 | 4.3 | 2.2 | 10 | 2.4 | 2.9 | 2.7 | 4.4 | 2.1 | 2.6 | 2.8 | 2.4 | 2.1 | 5.3 | 10.5 | 4.9 | | 2.9 | 4.3 | 3.6 | 1.2 | 3.4 | 2.8 | 0.8 | | 4.6 | 2.3 | 4.1 | 2.8 | 2.4 | 4.2 | 3.3 | 2 |
| **Alanine** | 376.7 | 290.5 | 243.7 | 307.9 | 519.8 | 185.2 | 190 | 224 | 226.1 | 314.8 | 262.1 | 292.7 | 340.7 | 161.7 | 272.6 | 826.8 | 276.1 | | 340.5 | 216.7 | 374.4 | 206 | 197.2 | 193.7 | 46.2 | | 247 | 254.6 | 502.2 | 247.5 | 299.9 | 275.5 | 289.2 | 380.2 |
| **Butyrate** | 10.7 | 10.7 | 12.4 | 9.2 | 29.5 | 2.2 | 9.1 | 6.2 | 6.6 | 5.9 | 8 | 5.8 | 12.4 | 6 | 8.2 | 24.2 | 6.9 | | 9 | 7.4 | 15.9 | 4.6 | 3.7 | 6.3 | 1.8 | | 5.2 | 5.2 | 15.7 | 6.7 | 6.5 | 7.4 | 8.1 | 8.2 |
| **Citrate** | 155.3 | 186.9 | 143.5 | 121.2 | 266.7 | 98.7 | 113.6 | 132.7 | 162.3 | 114.9 | 142.6 | 122.4 | 158.6 | 134.2 | 118.6 | 255.7 | 133.7 | | 208.8 | 117 | 158.5 | 112.7 | 156.8 | 127.8 | 26 | | 158.7 | 116.4 | 215.3 | 127.9 | 125.7 | 216 | 141.2 | 138.1 |
| **Creatine** | 50.1 | 42.8 | 50.5 | 38.3 | 73.6 | 10 | 47.9 | 52.2 | 50.4 | 36.7 | 47.2 | 49.4 | 49 | 45.4 | 40.6 | 116.6 | 42.4 | | 40.7 | 46.1 | 59.6 | 30.5 | 42.8 | 51.3 | 7.2 | | 50.4 | 38.3 | 80.6 | 46.8 | 43.1 | 63.9 | 56.5 | 0.8 |
| **Creatine phosphate** | 12.3 | 15.4 | 7.7 | 5 | 22.5 | 8.3 | 11.5 | 6.3 | 10.5 | 10.9 | 6.9 | 8.7 | 8.8 | 12 | 8.9 | 31.6 | 11.4 | | 17.7 | 10.1 | 12.7 | 6.8 | 9.6 | 13.5 | 1.1 | | 10 | 10.4 | 11.2 | 8.7 | 8.5 | 13.5 | 9.9 | 14.2 |
| **Creatinine** | 63.6 | 55.8 | 63.2 | 56.5 | 99.7 | 36.1 | 72.9 | 78.6 | 47.5 | 53.2 | 63.2 | 70.4 | 66.4 | 52.3 | 53.2 | 122.6 | 55.1 | | 61.9 | 61.9 | 80.3 | 40.4 | 36 | 65.8 | 7.5 | | 65.4 | 51.1 | 85.5 | 40.2 | 53.3 | 55.5 | 75.2 | 69.5 |
| **Dimethyl sulfone** | 4 | 5.9 | 3.6 | 3.2 | 7.5 | 1.2 | 3.2 | 3.7 | 4.2 | 1.5 | 2.6 | 3.6 | 2 | 3.1 | 3.2 | 5.5 | 7.9 | | 4.1 | 5.3 | 4.2 | 2.2 | 3.7 | 3.5 | 0.7 | | 6.2 | 3.2 | 6 | 4.5 | 4.2 | 6.7 | 2.7 | 2.8 |
| **Formate** | 26.4 | 15.6 | 19.3 | 21.2 | 36.9 | 16.2 | 21.4 | 21.6 | 15.4 | 19.7 | 14.7 | 19.4 | 27.4 | 21.5 | 14.3 | 45.6 | 26.4 | | 22.1 | 18.8 | 24.1 | 23.2 | 15.2 | 19.9 | 14.1 | | 18.1 | 21.6 | 28.6 | 16.5 | 19.9 | 14.4 | 17.2 | 15.8 |
| **Glucose** | 3206 | 2494.8 | 6748.9 | 2600.4 | 5948.3 | 1636 | 2856.2 | 2830.1 | 5671.3 | 2260.1 | 2663.1 | 2913.5 | 2641.6 | 2310.2 | 3061.5 | 6102.8 | 3199 | | 2201.6 | 3710 | 2765.3 | 2502.2 | 2162 | 3124.3 | 354.7 | | 4065.4 | 1982.6 | 3525.4 | 2616 | 2010.2 | 1899.7 | 2288.7 | 2929.8 |
| **Glutamine** | 372.2 | 208.6 | 204.2 | 288.9 | 471.6 | 230.5 | 308.7 | 304.7 | 268.2 | 239.4 | 300 | 289.5 | 279.6 | 337.6 | 276.7 | 425.8 | 439.7 | | 338.9 | 275.2 | 367.6 | 370.5 | 344.3 | 316.6 | 67.3 | | 276.4 | 372.7 | 436.7 | 376.6 | 346.4 | 393.1 | 258.3 | 311.1 |
| **Glycerol** | 267.6 | 205.8 | 170.2 | 207.4 | 411.7 | 138.6 | 154.8 | 243.9 | 208.4 | 200.6 | 206.8 | 224.5 | 209.4 | 241.1 | 199.9 | 653.1 | 165.4 | | 229.5 | 168.4 | 259.6 | 138 | 205 | 133.9 | 71 | | 136.1 | 135.6 | 341.1 | 204.4 | 175.7 | 234.5 | 199.1 | 261.5 |
| **Glycine** | 204.4 | 135.9 | 363.1 | 144.1 | 388 | 86.9 | 152.8 | 154.9 | 311.5 | 125.2 | 152.2 | 165.2 | 147.3 | 124 | 176.1 | 538.2 | 179.4 | | 119 | 202 | 158.6 | 136.4 | 121.3 | 174 | 19.3 | | 219.1 | 115.5 | 235.9 | 147.8 | 107.4 | 118.3 | 129.5 | 167.7 |
| **Histidine** | 50.4 | 47.9 | 32.5 | 34.9 | 59.1 | 41.2 | 48.2 | 33.9 | 42.2 | 36.3 | 41.8 | 40.3 | 42.8 | 32.1 | 40.6 | 69.4 | 58.3 | | 42.7 | 34.9 | 47.3 | 44.5 | 39 | 56.7 | 7.8 | | 55.6 | 36.9 | 61.2 | 41.4 | 45.3 | 42.4 | 42.6 | 45.5 |
| **Inosine** | 13.7 | 8.6 | 6.7 | 8.4 | 15.4 | 9.3 | 11.1 | 5.6 | 10 | 14.1 | 5.4 | 9.9 | 5.7 | 8.1 | 7.5 | 15.7 | 11.2 | | 9.6 | 9.2 | 10.3 | 7.8 | 11.2 | 6.5 | 0.8 | | 10.5 | 8.6 | 8.2 | 4.5 | 4.4 | 7.4 | 7.9 | 6.7 |
| **Isoleucine** | 54.1 | 30.7 | 29.1 | 26.5 | 63.7 | 20 | 41.4 | 27.5 | 23.4 | 38.8 | 28.3 | 43.5 | 32.6 | 25 | 32.3 | 83 | 47.4 | | 43.8 | 26.4 | 44.8 | 23.2 | 25.5 | 28.3 | 6 | | 27.6 | 26.3 | 50.9 | 27.9 | 26.9 | 29.9 | 28.8 | 25.7 |
| **Lactate** | 2112.9 | 1660.2 | 2193.3 | 1540 | 3810.9 | 821.5 | 1172.2 | 1850.2 | 1110.4 | 2209.8 | 1448.3 | 1652.4 | 1361.9 | 1248.6 | 1181.3 | 4135.8 | 1287.5 | | 1841.2 | 1264 | 2162.9 | 1650.1 | 1111.1 | 1113.2 | 338.6 | | 1214.5 | 1250.6 | 1721.6 | 1382.7 | 1406.6 | 2021 | 1216.1 | 2190.2 |
| **Leucine** | 82 | 51.9 | 47.9 | 40.7 | 99 | 41.8 | 76 | 49.1 | 48.7 | 58.8 | 51.2 | 79.1 | 56 | 45.6 | 59.3 | 110.7 | 86.9 | | 71.1 | 42.2 | 68.3 | 46.8 | 45.5 | 53.3 | 8.8 | | 57.6 | 56.1 | 77.1 | 59.6 | 56.7 | 56.2 | 52.6 | 43.6 |
| **Lysine** | 51.6 | 62.3 | 44.2 | 40 | 78.4 | 42.4 | 52.8 | 33 | 40.8 | 53.4 | 31.5 | 52.9 | 54.8 | 45.3 | 51.4 | 152.6 | 58 | | 71.4 | 51.7 | 64.3 | 32.8 | 45.1 | 64.8 | 10.4 | | 42.7 | 64.1 | 56.8 | 40.3 | 51.5 | 52.5 | 62.2 | 73 |
| **Malonate** | 24.6 | 36.3 | 19.8 | 23.2 | 51.3 | 12 | 39.6 | 27.4 | 21.3 | 27.6 | 13.2 | 29.2 | 15.9 | 13.9 | 22 | 38.6 | 34.1 | | 37.5 | 24.3 | 31.7 | 21.5 | 19.9 | 20 | 1.9 | | 31 | 26 | 25.7 | 18.5 | 18.4 | 24.3 | 26.5 | 26.7 |
| **Methionine** | 81.8 | 39.6 | 23.7 | 37.3 | 112.6 | 39.7 | 50.6 | 27.5 | 36.8 | 46.9 | 37.3 | 37.1 | 45.3 | 52.2 | 32.2 | 91.3 | 77.2 | | 42.8 | 28.7 | 37.9 | 58.9 | 44.3 | 57.3 | 5.3 | | 47 | 41.5 | 56.3 | 40.4 | 42.9 | 47.3 | 37.8 | 46.4 |
| **Methylsuccinate** | 21.7 | 14.2 | 19.2 | 15.4 | 36.4 | 11.4 | 16.1 | 23.4 | 12 | 14.8 | 12.8 | 14.5 | 18.9 | 11.8 | 15.1 | 34.4 | 13.1 | | 15.5 | 13.5 | 17.7 | 14.9 | 12.8 | 15.3 | 3 | | 15.4 | 14.9 | 22 | 11.7 | 13.8 | 13.7 | 12.4 | 12 |
| **N.N-Dimethylglycine** | 2.9 | 1.6 | 1.1 | 1.5 | 3.5 | 2 | 1.9 | 1.5 | 1.7 | 1.7 | 2 | 1.9 | 1.7 | 1.4 | 1.6 | 4.5 | 2.7 | | 1.8 | 1.2 | 2 | 2.2 | 1.7 | 1.8 | 0.2 | | 2 | 2.3 | 3.3 | 2 | 1.4 | 2.2 | 1.5 | 2 |
| **O-Acetylcarnitine** | 24.5 | 11.4 | 8 | 10 | 31.7 | 7.2 | 15.2 | 10.5 | 7.6 | 17.4 | 6.4 | 14.6 | 8 | 11.7 | 6.8 | 28.2 | 16 | | 15.4 | 10 | 13.9 | 12.5 | 10.4 | 10.1 | 1.7 | | 10.1 | 10.5 | 11.6 | 8.6 | 9.1 | 13.2 | 9 | 11.6 |
| **Phenylalanine** | 52.3 | 50.1 | 35.3 | 37.9 | 79.6 | 36.2 | 53.1 | 33.3 | 38.3 | 51.8 | 44.2 | 53.9 | 41.8 | 47.6 | 40.8 | 88.7 | 60.7 | | 56.4 | 37.7 | 45.9 | 44 | 50.8 | 47.4 | 8.2 | | 52 | 56.1 | 70.4 | 46.5 | 40.8 | 46.8 | 35 | 44.2 |
| **Pyroglutamate** | 33.4 | 20.8 | 17.1 | 22.3 | 44.9 | 14 | 20.2 | 10.5 | 29.8 | 39.5 | 16.9 | 26.5 | 20.5 | 19.3 | 17 | 37.6 | 23.1 | | 22.8 | 29 | 32.2 | 24.6 | 17.2 | 19.3 | 6.2 | | 12.8 | 22.2 | 53.3 | 22.7 | 24.6 | 16 | 20.1 | 22.9 |
| **Pyruvate** | 16.1 | 28.5 | 40.6 | 45.7 | 62.2 | 13.3 | 24 | 46 | 18 | 25.2 | 31.8 | 29.2 | 37.4 | 20.9 | 25.8 | 75.6 | 32.9 | | 43.1 | 22.7 | 77.5 | 19.6 | 18.4 | 22.9 | 10.5 | | 18.5 | 20.8 | 47 | 28 | 29.4 | 41.6 | 32.4 | 46.7 |
| **Sarcosine** | 5.6 | 2.2 | 2.2 | 2.6 | 12.5 | 2.8 | 5.1 | 3.1 | 3.1 | 4.8 | 2.5 | 3 | 16.2 | 2.1 | 4.4 | 10.8 | 4 | | 2.9 | 2.1 | 4.2 | 4.9 | 2.7 | 5.7 | 0.4 | | 3.6 | 4.4 | 4.3 | 2.1 | 12.7 | 1.8 | 3.3 | 2.5 |
| **Serine** | 117.2 | 106.1 | 49.5 | 63.1 | 198.2 | 98.1 | 80.4 | 51.3 | 66.9 | 85.1 | 78.7 | 77.4 | 84.8 | 131.1 | 74.1 | 251.7 | 130.8 | | 90.4 | 64.8 | 98.7 | 78.8 | 94 | 91.1 | 15.7 | | 91 | 103.7 | 148.3 | 82.2 | 99.9 | 95.6 | 79.4 | 129.2 |
| **Threonine** | 97.5 | 74.7 | 50.5 | 51 | 128.4 | 92.5 | 65.6 | 66 | 86.4 | 64.3 | 74 | 94.9 | 87.3 | 76.1 | 81.7 | 184.4 | 73.2 | | 82.8 | 50 | 68.2 | 102.8 | 80.6 | 58.3 | 10.7 | | 73.6 | 69.8 | 116.7 | 72.4 | 74.3 | 64.3 | 60.1 | 76.5 |
| **Tyrosine** | 39.1 | 32.7 | 20.1 | 23.6 | 56.5 | 21.4 | 42 | 22.4 | 30.2 | 24.6 | 27.5 | 34.3 | 31.5 | 35 | 33.1 | 66.3 | 33.3 | | 35 | 16.2 | 26.5 | 21.6 | 25.4 | 27.1 | 5.3 | | 33 | 23.7 | 48.5 | 29.4 | 26 | 30.6 | 34.3 | 29.3 |
| **Valine** | 134.4 | 82.7 | 83.4 | 62.5 | 169.6 | 67.1 | 102.5 | 82.8 | 74.3 | 102.5 | 87.3 | 114.7 | 97.4 | 76.1 | 103.6 | 200.1 | 120.4 | | 105.3 | 73.8 | 112.5 | 79.8 | 73.4 | 74.2 | 14.5 | | 80 | 88.3 | 134 | 88.1 | 76.5 | 86 | 80.6 | 67.3 |
| **Trans-4-Hydroxy-L-proline** | 16.2 | 20 | 14.5 | 31.6 | 15.9 | 19.6 | 24.5 | 30.4 | 22.6 | 28.4 | 9.5 | 23.4 | 18.3 | 17.6 | 9.8 | 20.4 | 31.3 | | 14.4 | 14.7 | 23.4 | 26.6 | 18 | 19.8 | 6.7 | | 21.4 | 44.8 | 18.6 | 18.6 | 27.8 | 23.7 | 14.7 | 14.6 |
| **1-Methylhistidine** | 20.6 | 16.5 | 10.9 | 13 | 27.4 | 12.6 | 23 | 11.9 | 14.4 | 18.7 | 14.2 | 14.5 | 15.7 | 12.7 | 12.1 | 20.7 | 16.5 | | 17.1 | 19.3 | 17.1 | 19.1 | 15.7 | 14.8 | 3.4 | | 16 | 13 | 13.3 | 11.1 | 17 | 13.4 | 16.9 | 13.1 |
| Legend: Conventional poultry byproduct meal (CPM-c); Hydrolyzed poultry byproduct meal (EHPM-c); Nuclear magnetic resonance spectroscopy (¹H NMR).  Serum metabolites were quantified using proton ¹H NMR. Values are presented as mean ± SD. Data were median-normalized, Pareto-scaled and analyzed using Student’s t-test, with statistically significant differences considered at P ≤ 0.05 | | | | | | | | | | | | | | | | | | | | | | | | | | | | | | | | | | |

| **Supplementary Table 4.** Variable Importance in Projection (VIP) scores of serum metabolites contributing to group discrimination in the OPLS-DA model | | |
| --- | --- | --- |
| **Metabolites (μM/L)** | **VIP [t]** | **VIP [ortho-t]** |
| 1-Methylhistidine | 2.22 | 0.27 |
| Valine | 1.97 | 0.4 |
| Acetate | 1.85 | 1.0 |
| Glycerol | 1.8 | 0.28 |
| Methionine | 1.71 | 0.59 |
| N,N-Dimethylglycine | 1.48 | 0.34 |
| Malonate | 1.34 | 0.55 |
| Creatine phosphate | 1.3 | 0.87 |
| Inosine | 1.11 | 1.33 |
| Glucose | 1.1 | 1.34 |
| Creatinine | 1.06 | 1.1 |
| Glutamine | 1.05 | 1.12 |
| Methylsuccinate | 1.04 | 1.36 |
| 2-Hydroxyisobutyrate | 1.02 | 0.14 |
| Phenylalanine | 1.0 | 0.64 |
| 3-Hydroxyisovalerate | 0.96 | 0.64 |
| Histidine | 0.9 | 1.29 |
| Tyrosine | 0.84 | 0.61 |
| Formate | 0.79 | 1.43 |
| Serine | 0.72 | 1.01 |
| Glycine | 0.66 | 1.29 |
| Butyrate | 0.65 | 0.54 |
| trans-4-Hydroxy-L-proline | 0.63 | 0.89 |
| Acetone | 0.54 | 1.27 |
| Isoleucine | 0.53 | 1.42 |
| 3-Hydroxyisobutyrate | 0.53 | 1.36 |
| Lysine | 0.52 | 0.91 |
| Pyroglutamate | 0.52 | 0.4 |
| Dimethyl sulfone | 0.44 | 1.27 |
| Lactate | 0.4 | 0.9 |
| Sarcosine | 0.39 | 0.66 |
| Alanine | 0.35 | 1.14 |
| Creatine | 0.33 | 1.17 |
| Pyruvate | 0.25 | 0.37 |
| Leucine | 0.2 | 1.51 |
| Citrate | 0.13 | 1.22 |
| 1,7-Dimethylxanthine | 0.06 | 1.25 |
| Threonine | 0.05 | 1.09 |
| O-Acetyl carnitine | 0.01 | 0.97 |
| Legend: Unsupervised principal component analysis (PCA) and supervised orthogonal partial least squares discriminant analysis (OPLS-DA) were applied to evaluate metabolomic differences between groups. PCA significance was assessed using PERMANOVA. In the OPLS-DA, variable importance in projection (VIP) scores ranked metabolites according to their contribution to the discrimination between treatments. Metabolites with VIP > 1.0 were considered the most relevant to the model separation | | |
